# Outpatient Treatment Guidelines of Gunshot Wound to the Hand and Wrist Resulting in an Open Fracture: Case Report

**DOI:** 10.7759/cureus.31130

**Published:** 2022-11-05

**Authors:** Taylor Brown, Petr Gaburak, John Hwang

**Affiliations:** 1 College of Medicine, Washington State University, Spokane, USA; 2 Orthopedic Surgery, Orthopedics Northwest, Yakima, USA

**Keywords:** penetrating wrist injury, wrist injury, penetrating hand injury, hand trauma, gunshot wound

## Abstract

The incidence of non-fatal gunshot wounds has significantly increased in the past decade. The current guidelines lack clarity in treatment of bullet wounds to the hand and wrist.

An 18-year-old male presented to the emergency department with a gunshot wound to the hand/wrist resulting in an open fracture. The entrance wound was clean without visible bone. No neurovascular damage was reported. The wound was irrigated with saline, and a sterile dressing and splint was applied in the emergency department. The patient was discharged the same day with oral antibiotics and an appointment with an orthopedic hand specialist. Three days after the injury, the patient was taken to surgery to treat a fracture of the radius and lunate. No internal fixation was required. The fracture and bullet fragments were removed, and the patient was discharged on the same day. The patient recovered to a full range of motion and no infection was acquired throughout treatment and healing.

The current guidelines for the treatment and management of nonfatal gunshot wounds to the hand and wrist are inconsistent resulting in unnecessary admittance to the hospital. Our paper provides a template for future cases allowing for outpatient treatment.

## Introduction

Gun-associated injuries in the United States are a major public health crisis that is associated with significant levels of morbidity, mortality, and economic cost to the healthcare system. Between 2014-2018, there were an estimated 535,000 non-fatal gun injuries in the country, showing a substantial increase in gun-related injuries in the United States [1]. 

While non-fatal gunshot injuries are increasing, the management guidelines for injuries to the hand are still unclear leading to an increased burden on orthopedic trauma services as well as the healthcare system as a whole [2]. Current protocols for the treatment of open fractures have vague recommendations regarding the timing and urgency of surgical intervention in the operating room leaving the decision up to the attending physician [3,4]. Furthermore, research has shown varying degrees of antibiotic use amongst physicians with some studies urging for prophylactic IV antibiotic use without clear evidence of the necessity for infection prevention [5,6]. Thus, the lack of concrete guidance leaves the decision for wound treatment and management up to the discrepancy of the attending resulting in potentially unnecessary admission of patients to the hospital.

We report a case of a non-fatal gunshot wound to the hand/wrist with successful outpatient treatment and management practices to provide clarity to the guidelines, especially in rural areas that lack personnel and resources.

## Case presentation

**Figure 1:**
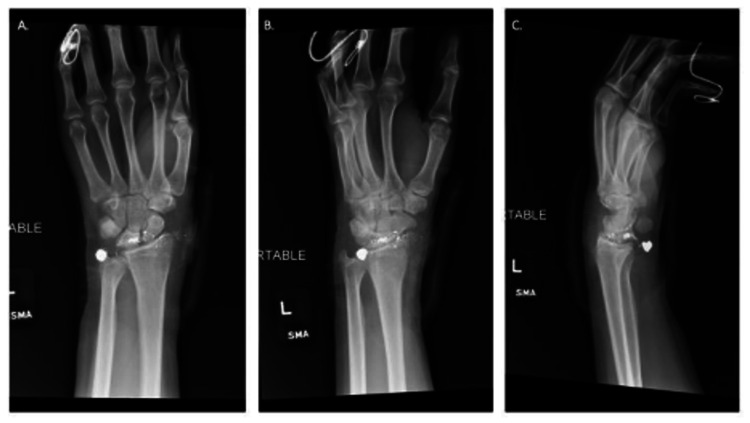
Preoperative plain radiographs. Anteroposterior and lateral X-Ray images (A-C).

An 18-year-old man with a gunshot wound to the left hand/wrist was brought to the emergency department. The injury was from a pistol of unknown caliber. The bullet had entered the radial aspect of the wrist, resulting in a fracture of the radius and lunate. The bullet broke into multiple fragments in the volar radiocarpal capsule, while the main fragment was lodged in the ulnar tunnel. A CT and x-ray were performed in the emergency department, confirming the above finding (Figure 1). The entrance wound was clean without exposure of the bone, tendon, or neurovascular structures. The patient was reported to have intact neurovascular function of the hand. The wound was irrigated with saline solution in the emergency department, and a sterile dressing and splint were applied. A tetanus vaccine was not administered, as the patient was up to date on his immunization. He was discharged from the emergency department with a follow-up appointment with an orthopedic hand specialist. The patient was prescribed oral Cephalexin 500mg four times daily for one week. 

**Figure 2:**
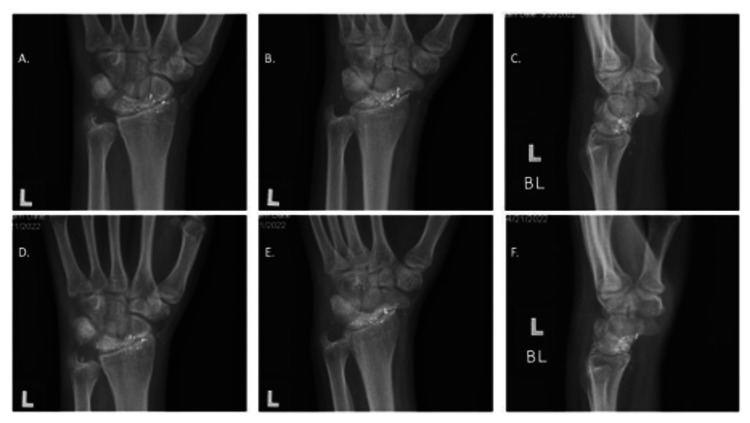
Postoperative plain radiographs. 10 days post-op anteroposterior and lateral X-Ray images (A-C). 5 week post-op anteroposterior and lateral X-Ray images (D-F).

The patient was evaluated by the orthopedic hand specialist two days after the injury and was scheduled for outpatient surgery the next day. The patient was brought to the operating room three days after the injury. The surgical exploration showed that the wound was clean without signs of infection. The bullet caused an intra-articular lunate and distal radius fracture involving less than 20% of the joint surface without major structural damage. It was determined that the patient did not require internal fixation for both the injured radius and lunate. The fracture fragments and bullet fragments were removed with partial excision of capsules and decompression of the ulnar nerve. Some small fragments were left inside the wrist as they were diffusely embedded in volar capsules but did not invade the joint space. The patient was discharged on the same day of the surgery. The hand was immobilized for two weeks and the patient began formal therapy afterward. He continued oral antibiotics for another week after surgery. X-ray images were obtained at 10 days and five weeks post-surgery to monitor wound healing (Figure 2). Five months after surgery, the patient had a nearly full range of motion and strength, with no pain or infection through the healing process. 

## Discussion

Gun violence has been steadily increasing over time; however, guidelines for the treatment and management of low-velocity gunshot wounds have not been concretely established [3,7]. This absence causes an increased strain on hospital systems that may be lacking resources and bed space. Our report provides an example of routine treatment and management protocol for a non-fatal gunshot wound to the hand and wrist to be used as a template for future care with a similar case. 

Gunshot wounds have been categorized based on the speed of the bullet into low-velocity (pistols) injuries and high-velocity (rifles) injuries. Our focus is on low-velocity gun injuries which are described as wounds from a bullet that moves at a speed of 2000 ft/s or less [8]. Previous theories believed the heat and speed of the bullet in low-velocity guns created a sterile environment that rarely led to infections [9]. However, a study conducted by Wolf et al. showed that bullet wounds are not as sterile as was theorized [10]. This evidence then prompted further investigation of proper guidelines for wound care and infection prevention. 

Initial wound care according to multiple guidelines suggests the sterilization of the gunshot wound with a saline rinse. However, the need for immediate surgical debridement is still being debated. In addition, the traditional recommendation of intravenous antibiotics for 24 hours for such injury has been in question. Several studies have shown that simple irrigation with minimal debridement has reported low rates of infection [11,12]. Due to the low rates of infection, prophylactic antibiotic use has been a topic of discussion. 

Many studies currently recommend prophylactic treatment of gunshot wounds with IV antibiotics, thus leading to patients being admitted to the hospital [5,13,14,15]. However, the evidence on the efficacy of IV antibiotic use compared to oral antibiotic use has shown no difference [16]. Furthermore, the rates of infection of antibiotic use versus no antibiotic use have shown no statistical significance. However, many of these studies lack statistical power [11]. Although the evidence regarding antibiotic use is currently being debated, most surgeons continue to prescribe antibiotics prophylactically for gunshot wound patients despite having no concrete guidelines from institutions [6]. Finally, the patient should also be evaluated for their tetanus vaccination status, and if out of date, be provided with prophylactic treatment [3].

Lastly, the timeline for surgical care is critical in maintaining normal functioning and mobility. Current emergency department protocols are not established, thus leaving the discretion of surgical urgency to the attending and potentially resulting in unnecessary admittance of patients. Several studies have shown that simple wound care and management in the emergency department are sufficient treatment until they are seen in an outpatient clinical setting [8,12,17]. Surgical intervention should be done within one week to maximize restoration of function and mobility of the hand/wrist [18].

Based on the case report and the evidence presented, we recommend initial wound management with saline irrigation without immediate surgical debridement. Following wound care, splinting of the hand/wrist is sufficient stabilization. Due to inconclusive evidence concerning antibiotic use, we recommend following a conservative method with oral antibiotics upon discharge of the patient. If the tetanus vaccine is not up to date, provide prophylactic treatment. A referral should be sent for appropriate specialty services and, if needed, surgery could be done in a week without compromising the prognosis for the patient.

Our paper addresses outpatient treatment for an open fracture of the hand/wrist with minimal soft tissue damage caused by a low-velocity, non-fatal gunshot injury. Our paper does not account for neurovascular compromise, extensive soft tissue injury, or high-velocity gunshot wounds. Future studies should investigate whether this outpatient treatment can be applied to cases with more extensive damage to the hand and/or wrist. Patient risk factors should also be assessed concerning prophylactic antibiotic use.

## Conclusions

Given the lack of a distinct protocol for the treatment of non-fatal gunshot wounds to the hand/wrist, we urge further evaluation and revision of current guidelines. We recommend the utilization of an outpatient management protocol for similar cases consisting of flushing out the wound, prescribing oral antibiotics, tetanus prophylactic treatment if needed, and splinting the hand/wrist in the emergency department until surgery in an outpatient clinical setting. Utilization of this outpatient management protocol of low velocity, non-fatal gunshot wounds to the hand may be helpful to prioritize resources and beds, especially in low-resource areas such as rural healthcare systems.
